# Duplex Telomere-Binding Proteins in Fungi With Canonical Telomere Repeats: New Lessons in the Rapid Evolution of Telomere Proteins

**DOI:** 10.3389/fgene.2021.638790

**Published:** 2021-02-26

**Authors:** Neal F. Lue

**Affiliations:** ^1^Department of Microbiology and Immunology, W. R. Hearst Microbiology Research Center, New York, NY, United States; ^2^Sandra and Edward Meyer Cancer Center, Weill Medical College of Cornell University, New York, NY, United States

**Keywords:** telomeres, TRF1 and TRF2, BLM, Tay1, *Ustilago maydis*, Basidiomycota, Ascomycota

## Abstract

The telomere protein assemblies in different fungal lineages manifest quite profound structural and functional divergence, implying a high degree of flexibility and adaptability. Previous comparative analyses of fungal telomeres have focused on the role of telomere sequence alterations in promoting the evolution of corresponding proteins, particularly in budding and fission yeast. However, emerging evidence suggests that even in fungi with the canonical 6-bp telomere repeat unit, there are significant remodeling of the telomere assembly. Indeed, a new protein family can be recruited to serve dedicated telomere functions, and then experience subsequent loss in sub-branches of the clade. An especially interesting example is the Tay1 family of proteins, which emerged in fungi prior to the divergence of basidiomycetes from ascomycetes. This relatively recent protein family appears to have acquired its telomere DNA-binding activity through the modification of another Myb-containing protein. Members of the Tay1 family evidently underwent rather dramatic functional diversification, serving, e.g., as transcription factors in fission yeast while acting to promote telomere maintenance in basidiomycetes and some hemi-ascomycetes. Remarkably, despite its distinct structural organization and evolutionary origin, a basidiomycete Tay1 appears to promote telomere replication using the same mechanism as mammalian TRF1, i.e., by recruiting and regulating Blm helicase activity. This apparent example of convergent evolution at the molecular level highlight the ability of telomere proteins to acquire new interaction targets. The remarkable evolutionary history of Tay1 illustrates the power of protein modularity and the facile acquisition of nucleic acid/protein-binding activity to promote telomere flexibility.

## Introduction

Linear eukaryotic chromosome ends are stabilized by protein assemblies that organize the repetitive terminal DNA sequence (∼5–20 base pairs per repeat unit) into protective structures that are resistant to aberrant degradation and recombination (O’Sullivan and Karlseder, 2010; Jain and Cooper, 2011; de Lange, 2018). The DNA component of this protective “cap,” known as telomeres, usually consists of a duplex region of hundreds to thousands of nucleotides and a 3′ overhang of tens to hundreds of nucleotides (also named the G-tail because of its G-rich nucleotide composition). Both the duplex region and the G-tail consist of the same short repeat unit, and both are bound by sequence-specific recognition proteins, which in turn recruit other proteins crucial for telomere protection. Collectively these proteins suppress the action of checkpoint and repair factors that can engender profound genomic instability.

Besides telomere protection, the other major function of telomere-bound proteins is to help preserve and replenish telomere DNAs. Despite their fundamental importance, telomere DNAs are subjected to progressive attrition owing to incomplete end replication (Olovnikov, 1996; Lansdorp, 2005). Telomeres can also experience drastic truncation due to recombinational excision or replication fork collapse (Lustig, 2003; Lansdorp, 2005). To compensate for such losses, eukaryotic cells employ telomerase and the primase-pol α complex to extend the G-tail and the complementary C-strand of telomeres, respectively (Autexier and Lue, 2006; Blackburn and Collins, 2011; Pfeiffer and Lingner, 2013; Lue et al., 2014). In addition, the cells are known to recruit a number of DNA helicases and repair proteins to overcome or alleviate problems arising from telomere replication fork stalling or collapse (Martinez and Blasco, 2015). Not surprisingly, these DNA maintenance pathways are under robust control by telomere-bound proteins in order to maintain telomere lengths within a size range that is optimal for telomere function.

Even though one might have imagined that the crucial importance of telomeres would make the nucleoprotein structures highly conserved in evolution, telomeres have in fact been subjected to rapid evolution, especially in selected clades. Nowhere is this malleability more evident than in the fungal phyla that include as their members some the most frequently employed model organisms. In both Saccharomycotina and Taphrinomycotina, which include *Saccharomyces cerevisiae* and *Schizosaccharomyces pombe*, respectively, the telomere DNA repeat sequence are often irregular and variable, and they differ substantially from the canonical sequence 5′-TTAGGG-3′/5′-CCCTAA-3′ (Steinberg-Neifach and Lue, 2015; Sepsiova et al., 2016). While the underlying reasons for such telomere DNA sequence divergence remain obscure, it does highlight the adaptive capacity of fungal cells to stabilize the altered sequence at chromosome ends.

In contrast to the budding and fission yeasts, many other fungi in the Ascomycota phylum, including most of the filamentous fungi, have retained the canonical TTAGGG sequence. This telomere sequence is also widely conserved in the more basal branches of fungi such as Basidiomycota and Mucoromycota. To what extent the telomere assemblies in these “non-standard” fungi manifest structural and functional divergence is an open question. Indeed, while putative telomere-binding proteins can be readily identified in many such fungi, very few studies have experimentally interrogated the functions of these proteins. Despite this substantial knowledge gap, a few recent studies have begun to provide tantalizing hints of significant structural and functional divergence at basidiomycetes telomeres (Kramara et al., 2010; Yu et al., 2018, 2020). In particular, it appears that even in the context of an invariant telomere repeat sequence, a new family of telomere DNA-binding protein can emerge and acquire telomere functions through the acquisition of new DNA sequence specificity and protein partners. It can also acquire non-telomeric functions, or be lost in some descendants. The potential of telomeres to evolve new regulatory mechanisms is thus not confined to scenarios that entail DNA sequence alterations.

In this focused review, I will first provide a very brief overview of Ascomycota telomere variability, highlighting the well characterized, co-evolving telomere DNA sequence and recognition proteins in this phylum. This will be followed by a more in-depth discussion of recent works on telomere regulations in *Ustilago maydis*, a member of the Basidiomycota phylum. A special emphasis will be on the *U. maydis* Tay1 protein, which appears to belong to a relatively new protein family in fungal evolution. Members of Tay1 are confined to Ascomycota and Basidiomycota, and while they all bind the 5′-TTAGGG-3′/5′-CCCTAA-3′ repeat unit with high affinity and sequence specificity, these proteins evidently mediate distinct telomeric and non-telomeric functions in different fungi. Notably, the *U. maydis* Tay1 protein, despite being structurally different from mammalian TRF1 (a major double strand telomere binding protein), exhibits surprising mechanistic and functional similarities to this mammalian protein. The origin of Tay1 and the implications of Tay1 diversity for the malleability and adaptability of telomeres are discussed.

## Organisms With Canonical and Variant Telomere Repeats: Recognition of Double-Stranded Telomeres by Distinct Myb-Containing Proteins

**FIGURE 1:**
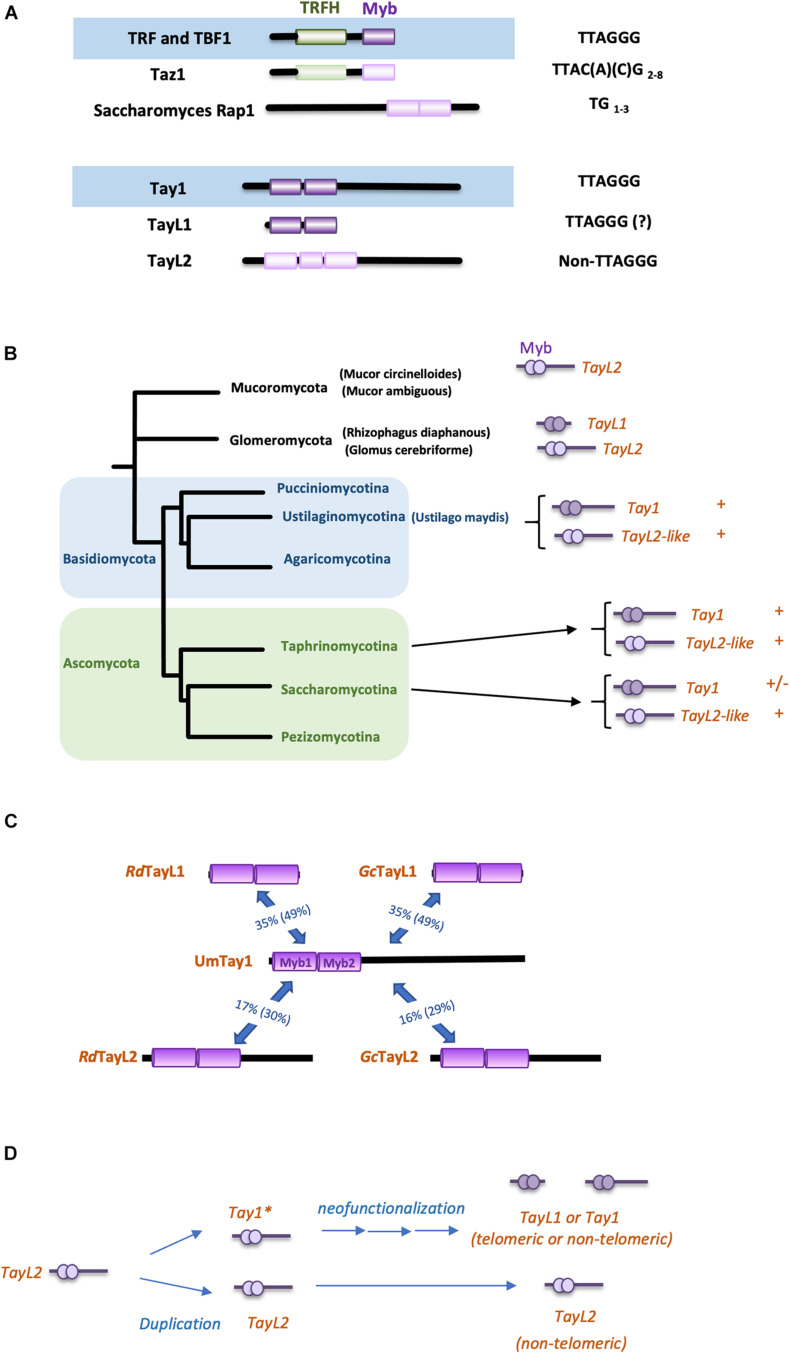
Duplex telomere binding proteins and their evolution in fungi. **(A)** The distinctive structures of duplex telomere binding proteins and their homologs (top). The domain structures of the major duplex telomere-binding proteins in vertebrates (TRF1 and TRF2), fission yeast (Taz1), and budding yeast (Rap1) are displayed. Both TRF1 and TRF2 consist of an N-terminal dimerization and protein-protein interaction domain (TRFH) and a C-terminal DNA binding motif (Myb). Taz1 has a similar domain structure as TRF1/2, but with a variant TRFH and a variant Myb domain. Rap1 contains two variant Myb motifs near its C-terminus. The sequences recognized by these proteins are shown to the right. (bottom) The domain structures Tay1, TayL1, and TayL2 are illustrated. Tay1 is a fungus-specific telomere-binding protein with two consecutive Myb motifs neat its N-terminus. These two Myb motifs exhibit strong similarities to vertebrates TRF1 and TRF2, and specifically bind the TTAGGG repeats. TayL1 resembles Tay1 but does not contain the C-terminal, non-Myb region found in Tay1. TayL2 has a similar domain structure as Tay1, but carries more divergent Myb motifs that are not predicted to bind TTAGGG. In some annotations of TayL2 homologs, this protein is postulated to contain a third Myb motif in between the two motifs that align to the Tay1 protein. The sequences recognized or presumed to be recognized by these proteins are shown to the right. **(B)** The distribution of Tay1, TayL1, and TayL2 in fungi. The distributions of Tay1, TayL1, and TayL2 in various fungal phyla are displayed. TayL2 is widely disseminated in Basidiomycota and Ascomycota, and is present in neighboring fungal branches including Glomeromycota and Mucoromycota. Tay1 is also widely disseminated in Basidiomycota and Ascomycota, but often absent in Saccharomycotina. TayL1 is apparently restricted to Glomeromycota. **(C)** The degrees of sequence similarities between the Tay1, TayL1, and TayL2 protein families. The extent of sequence identity and similarity (in parenthesis) between the Myb motifs of *Um*Tay1 and various TayL1 and TayL2 homologs are displayed. *Rd* and *Gc* designate *Rhizophagus diaphanous* and *Glomus cerebriforme*, respectively. Both belong to the Glomeromycota phylum. **(D)** Model for the origin and subsequent evolution of Tay1. A common ancestor of Tay1 and TayL1 (Tay1*, with high affinity and sequence specificity for TTAGGG) is hypothesized to emerge through gene duplication and modification of a Tay1-like gene such as TayL2. Subsequently, Tay1* may evolve into either the miniaturized TayL1 in Glomeromycota, or Tay1 in Basidiomycota and Ascomycota, and acquire either telomeric or non-telomeric functions. See main text for more details.

The most prevalent telomere repeat unit and possibly the most ancient is 5′-TTAGGG-3′/5′-CCCTAA-3′, which is found in various fungi, plant, metazoans, and protozoa. In organisms with this telomere repeat unit, the duplex region is typically recognized directly by a member of the TRF protein family, whereas the G-tail is bound by an OB-fold protein named POT1 (de Lange, 2018; Cicconi and Chang, 2020). In most mammalian cells, two structurally similar TRF homologs (TRF1 and TRF2) play partially overlapping and non-redundant functions in telomere protection and telomere maintenance (de Lange, 2018). Deleting or depleting TRFs often triggers significant telomere length alterations as well as structural abnormalities. TRF homologs are bi-partite proteins that consist of an N-terminal TRFH domain responsible for dimerization and a C-terminal Myb motif responsible for DNA-binding (Figure 1A). TRFs also employ multiple surface features within and outside the TRFH domain to interact with partners that regulate telomere functions (Chen et al., 2008; Kim et al., 2009). For example, mammalian TRF1 is thought to utilize a basic patch (located in between its TRFH and Myb domain) to bind and recruit BLM helicase, which in turn promotes the complete replication of telomeres by unwinding G-rich replication barriers (Lillard-Wetherell et al., 2004; Sfeir et al., 2009; Zimmermann et al., 2014). Similarly, mammalian TRF2 has been shown to recruit another helicase (RTEL1) as well as replisome proteins (Claspin, DONSON, etc.) to facilitate telomere replication (Sarek et al., 2015, 2019; Rai et al., 2019; Cicconi and Chang, 2020). However, unlike TRF1, TRF2 is responsible for a key protective function of telomeres by virtue of its ability to suppress telomere-telomere fusions.

Notably, the TRF proteins possess a variant of the Myb motif that is highly specific for the canonical TTAGGG sequence (Court et al., 2005). Hence, in budding and fission yeasts, where the telomere repeat units are made up of different sequences, the closest TRF homologs cannot recognize the telomere repeats with high affinity and do not function as the main duplex telomere-binding proteins. Instead, budding and fission yeasts utilize two other Myb-containing proteins, named Rap1 and Taz1, to coat and protect their respective telomeres (Buchman et al., 1988; Lustig et al., 1990; Cooper et al., 1997; Steinberg-Neifach and Lue, 2015; Sepsiova et al., 2016). In contrast to Rap1 and Taz1, the most TRF-like genes in budding and fission yeasts (often named Tbf1) provide important functions not at telomeres, but at subtelomeres or elsewhere in the genome. *S. cerevisiae* Tbf1, for example, regulates subtelomere structure and function, while also acting as a transcription factor upstream of various snoRNA genes (Fourel et al., 1999; Koering et al., 2000; Preti et al., 2010). In addition, *Sc*Tbf1 has been shown to regulate double strand break repair (Bonetti et al., 2013). These diverse and non-telomeric functions of budding yeast Tbf1 support the notion that this protein shares a common ancestry with TRFs, but has evolved and maintained functions away from the telomere terminal repeats due to the divergence of telomere sequence in this organism. Together these observations on Rap1, Taz1, and Tbf1 underscore one specific mechanism that promotes the remodeling of the telomere nucleoprotein complex.

## Fungi With Ttaggg Repeats: How Prone Are They to Telomere Nucleoprotein Remodeling?

As illustrated in the preceding section, the flexibility of the fungal telomere complex is evident in sub-phyla that experienced substantial telomere sequence divergence. Many fungi in the more basal clades, however, have retained the canonical, 6-bp telomere repeat. An interesting question, then, is whether the telomere proteins in these clades might exhibit less flexibility and less remodeling. While many genomes in these clades have been sequenced and putative telomere-binding proteins can be readily identified (see e.g., Sanchez-Alonso and Guzman, 2008), there are as yet, very few studies that experimentally interrogate the functions of these proteins. The best studied organism in this regard is *U. maydis*, a plant pathogen that forms corn galls. This yeast-like fungus was developed by Robin Holliday several decades ago as a model system for studying recombinational repair (Holloman et al., 2008). It belongs to the Basidiomycota phylum, members of which also include the human pathogen *Cryptococcus neoformans*. As a model for telomere research, *U. maydis* offers a number of advantages beyond the standard budding and fission yeast model, including (i) its greater resemblance to the mammalian system with respect to the recombination and repair machinery (Holloman et al., 2008), and (ii) its retention of the same telomere repeat as the mammalian repeat (Guzman and Sanchez, 1994; Sanchez-Alonso and Guzman, 1998). Interestingly, with regard to shelterin-like telomere-binding proteins in the *U. maydis* genome, several initial surveys revealed a putative Pot1 ortholog, but nothing resembling the mammalian TRF proteins (Sanchez-Alonso and Guzman, 2008; Yu et al., 2013). Instead, a protein bearing consecutive Myb motifs near its N-terminus (named *Um*Tay1 or *Um*Trf1) was postulated to be the most plausible candidate for binding the double-stranded region of telomeres (Yu et al., 2018, 2013). Notably, in addition to having N-terminal Myb motifs, *Um*Tay1 differs from a standard TRF homolog in having a much larger size (∼150 kD) and in lacking a TRFH dimerization domain (Figure 1A).

These initial conjectures notwithstanding, a TRF/TBF-like gene (named *Um*Tbf1 or *Um*Trf2) was subsequently uncovered in *U. maydis*, suggesting that this fungus may harbor two structurally distinct duplex telomere binding proteins (Sepsiova et al., 2016; Yu et al., 2020). To simplify the discussion, I will henceforth refer to these two proteins as *Um*Tay1 and *Um*Trf2, respectively.

Experimental interrogation of *Um*Tay1 and *Um*Trf2 confirmed the roles of both proteins in telomere regulation and revealed an interesting division of labor that is different from other systems (Yu et al., 2020). *Um*Tay1, on the one hand, plays minimal roles in telomere protection — deletion of the gene triggers neither growth defects nor telomere structural abnormalities. Further analysis of *Umtay1Δ* revealed preferential loss of long telomeres and the suppression of telomere recombination in the context of *ku70* transcriptional repression, i.e., Ku70 depletion. These phenotypes are reminiscent of *U. maydis* mutants with a knockout of *blm*, a conserved helicase with functions in both DNA repair and telomere regulation. Previous studies have shown that the *Um*Blm helicase positively stimulates both telomere replication and telomere recombination (Yu et al., 2015, 2018). Indeed, *Um*Tay1 physically interacts with Blm and modulates Blm helicase activity in a substrate sequence-dependent manner (Yu et al., 2020). Thus, by regulating the Blm helicase activity, *Um*Tay1 appears to perform similar functions in these two pathways. In contrast to *Um*Tay1, *Um*Trf2 plays a pivotal role in telomere protection: deletion of *trf2* is lethal, and transcriptional repression of this gene triggers a constellation of telomere aberrations that are indicative of de-protection, including telomere length heterogeneity, accumulation of ssDNA and extra-chromosomal telomere repeats (Yu et al., 2020). Therefore, despite having similar affinity and binding specificity for double-stranded telomere repeats, *Um*Tay1 and *Um*Trf2 mediate largely non-overlapping functions in telomere regulation. Notably, this division of labor is somewhat different from that in mammals, where two structurally similar proteins (i.e., TRF1 and TRF2) execute broadly related functions. For example, while TRF1 is known to play a critical and preferential function in telomere replication by recruiting BLM helicase to unwind G-rich structural barriers (Sfeir et al., 2009; Zimmermann et al., 2014), TRF2 also makes notable contributions by interacting with the RTEL1 helicase as well as replisome-associated proteins (Kim et al., 2009; Sarek et al., 2015; Rai et al., 2019; Cicconi et al., 2020). In addition, even though TRF2 appears to be the main mediator of telomere protection by suppressing ATM activation and telomere fusions, TRF1 also contributes to chromosome end protection (de Lange, 2018; Lee et al., 2018). The structural and functional differences between the duplex telomere binding proteins in fungi and mammals indicate that the retention of the same telomere repeat does not preclude the evolution of new telomere regulatory factors or functional shuffling among proteins capable of binding telomere repeats.

## The Evolutionary Origin of the Tay1 Protein Family

Given its structural resemblance to TRF/TBF, *Um*Trf2 most likely shares a common ancestry with prototypical TTAGGG-binding proteins, and has inherited its telomere functions from the same ancestor as TRF1/2. The origin of Tay1 is less clear. Bioinformatic analysis revealed closely related family members with tandem Myb motifs in the Basidiomycota and Ascomycota phyla, but not in more basal lineages such as Mucoromycota and Glomeromycota (Figure 1B). Thus, Tay1 may have originated through gene duplication and modification from a similar protein in the common ancestor of all of these fungal branches. In other words, the antecedent of Tay1 may be a structurally similar protein shared by Basidiomycota, Ascomycota, and the more basal phyla.

Based on this rationale, I performed psi-BLAST screening of Tay1-like proteins in Glomeromycota and Mucoromycota (the closest fungal phyla that occupy a more basal position than Basidiomycota and Ascomycota), and analyzed statistically significant hits with respect to (i) the presence of tandem Myb domains; and (ii) the conservation of putative TTAGGG repeat-binding residues (Court et al., 2005; Yu et al., 2020). Two protein families with the highest sequence similarity scores were uncovered in psi-BLAST and were named TayL1 (Tay1-like 1) and TayL2, respectively (Figure 1B). Members of these two families are variably distributed in the two analyzed fungal clades. Glomeromycota, for example, harbors both TayL1 and TayL2, whereas Mucorales appears to harbor only TayL2. TayL1, a small protein that comprises just two copies of the Myb domain, is the prime candidate for being the closest relative of Tay1, given their notable sequence similarities (∼35% identity, ∼50% similarity, and ∼5% gaps in the aligned Myb domain region) (Figures 1B,C). TayL1 also appears to share almost all amino acids residues in TRF that are implicated in binding the TTAGGG repeats (Supplementary Figure 1), supporting its ability to recognize this sequence. Whether TayL1 actually executes a telomere function is an interesting question for future investigation. In contrast, TayL2, a much larger protein (∼550–700 amino acids) with extra-Myb regions, appears to be more distantly related to Tay1 (∼17% identity, ∼30% similarity, and ∼15% gaps in the aligned Myb domain region), and its lack of several putative TTAGGG-binding residues renders its potential for a telomere function less plausible (Supplementary Figure 1). Indeed, a couple of TayL2-like proteins (UMAG 04101 and UMAG 10544), distinct from *Um*Tay1, can also be discerned in the genome of *U. maydis* and those of other Basidiomycota/Ascomycota species, indicating that this represents a distinct protein family with an ancient evolutionary history. (Some of the TayL2 entries in the database are annotated as Bas1-like because of their similarities to the transcription factor Bas1 in *S. cerevisiae*. However, *S. cerevisia*e Bas1 has much weaker sequence similarity to *Um*Tay1 than the TayL2s identified in the psi-BLAST analysis). It is also worth noting while psi-BLAST identified two Myb motifs in TayL2 that align well to the DNA-binding region of Tay1, the annotations of multiple TayL2s in the databases postulate the existence of a third Myb motif, which is not present in Tay1 and which further underscores the greater evolutionary distance between these two protein families (Figure 1A).

Taken together, the distribution of Tay1-like proteins can be used to construct a parsimonious model for their evolutionary kinship, as follows (Figure 1D). Prior to the divergence of Mucoromycota from Basidiomycota and Ascomycota, Tay1^∗^ (common ancestor of Tay1 and TayL1) emerged as a TTAGGG repeat binding protein, possibly through duplication and evolutionary tinkering of another protein with tandem Myb domains (e.g., TayL2). Subsequently, in some sub-lineages of Basidiomycota and Ascomycota, this protein acquired telomere function through its ability to localize to telomeres and interact with other telomere regulators such as Blm. However, in most Mucoromycota species, this gene duplication and neofunctionalization never transpired, and TAY1/TAYL1 either remained a protein with minimal telomere function, or was lost from the genome. It is worth noting that an underlying factor that enables this evolutionary scenario is the modular nature of proteins, which in this case allows the ancient Tay1 to acquire the necessary DNA-binding and telomere-regulatory activities in a step-wise fashion.

## Discussion: Implications for the Evolution of Tay1 and Protein-Protein Interactions at Telomeres

The foregoing discussion argues for a relatively recent origin for Tay1 through alteration of another protein that did not possess telomere-binding activity or telomere function. As such, Tay1 emerged in an organism where the telomere functions are presumably well served by a TRF-like protein (e.g., the ancestor of *Um*Trf2). Any telomere function acquired by Tay1 in this context was likely to be non-essential or redundant – as evident from the phenotypes of the *Umtay1Δ* mutant. These conjectures have ramifications for the subsequent evolution of Tay1 in basidiomycetes and ascomycetes. In particular, given that there was no strong selection pressure for the telomere function(s) of ancient Tay1, this protein would have been relatively unconstrained in adopting other cellular functions. Indeed, the *S. pombe* Tay1 homolog, also known as *Sp*Teb1, has been shown to regulate transcription rather than telomeres (Valente et al., 2013). The ancient Tay1 could also be lost from the genome without great detriment –unless, of course, it had somehow acquired a more critical function. Indeed, no Tay1 homolog can be readily identified in most of the Saccharomycotina yeasts, including the *Kluyveromyces*, *Saccharomyces*, and *Candida* species. As noted before, these yeasts have experience drastic telomere sequence alterations that eliminated the canonical TTAGGG repeats from chromosome ends. Hence any Tay1 that was present in the ancestor of these yeasts would have been unable to remain telomere-bound in the descendants. Unless this Tay1 has somehow acquired critical non-telomeric functions, there would be little selection pressure for its retention. An interesting exception to this evolutionary scenario is *Yarrowia lipolytica*, an early branching Saccharomycotina yeast. Tay1 appears to be essential in this yeast, and deleting just one TAY1 allele in a diploid strain causes drastic telomere shortening, supporting an important function in telomere maintenance (Kramara et al., 2010). The distinct fate of *Yl*Tay1 can be understood in light of the mild telomere sequence alteration that transpired in this yeast; while the telomere repeat in *Y. lipolytica* deviated from the canonical repeat (TTAGTCAGGG rather than TTAGGG), the retention of the GGGTTA core element recognized by the Myb motif allowed *Yl*Tay1 to remain telomere-bound and to perform its telomere maintenance function (Kramara et al., 2010). Thus, the presence or absence of Tay1, as well as its divergent functions in different fungi, can be largely rationalized by its evolutionary origin and its DNA recognition property.

Given that *Um*Tay1 has a fundamentally different structure and evolutionary origin from mammalian TRF1, it is perhaps surprising that these two proteins share the same molecular partner (Blm) and the same telomere function (promoting replication). This apparent instance of convergent evolution at the molecular level suggests that telomere proteins may be quite adapt at evolving new protein-protein interactions. The greater propensity of telomere proteins to acquire interaction partners is also consistent with the growing list of proteins shown to bind shelterin subunits, especially TRF1 and TRF2. Both TRF1 and TRF2 contain within its TRFH domain a surface groove capable of binding a short peptide motif (TBM; Y/F/H-X-L-X-P) (Chen et al., 2008). An impressive list of DNA repair and replisome proteins have been shown to carry this motif, and to make functionally important interaction with TRF1 or TRF2 (Chen et al., 2008; Kim et al., 2009; Wan et al., 2013; Cicconi et al., 2020). Notably, this motif is not reliably conserved in evolution in mammals (e.g., Cicconi et al., 2020), suggesting that individual TRF-target interaction can be gained or lost quite recently. This again echoes the notion that telomere proteins are quite facile in evolving new interaction partners. Could there be some unique feature of telomeres that facilitate this? One possibility is the repetitive nature of the telomere sequence, which results in the clustering (and increased local concentration) of telomere proteins. In this setting, even a mutation that results in low affinity binding to a novel partner may be sufficient to support enough complex formation to result in a selectable phenotype. This is similar to a previous proposal that emphasizes the power of protein co-localization to drive evolutionary changes (Kuriyan and Eisenberg, 2007). Thus it would not be surprising if future studies of telomere regulation in different organisms were to uncover more examples of convergent evolution at the molecular level. A possible theme is that the key players in telomere regulation (e.g., Blm) will turn out to be well conserved in evolution but the molecular interactions through which their functions are executed may not be.

## Author Contributions

NL performed the bioinformatic analysis and wrote the manuscript.

## Conflict of Interest

The author declares that the research was conducted in the absence of any commercial or financial relationships that could be construed as a potential conflict of interest.
