# Supplementary material for: Duplex Telomere-Binding Proteins in Fungi With Canonical Telomere Repeats: New Lessons in the Rapid Evolution of Telomere Proteins
Source: Front Genet. 2021 Feb 26;12:638790. doi: 10.3389/fgene.2021.638790 (PMC7952879; doi:10.3389/fgene.2021.638790)
Supplement: Supplementary file 1 [file Data_Sheet_1.pdf]

Supp. Fig. 1

Lue

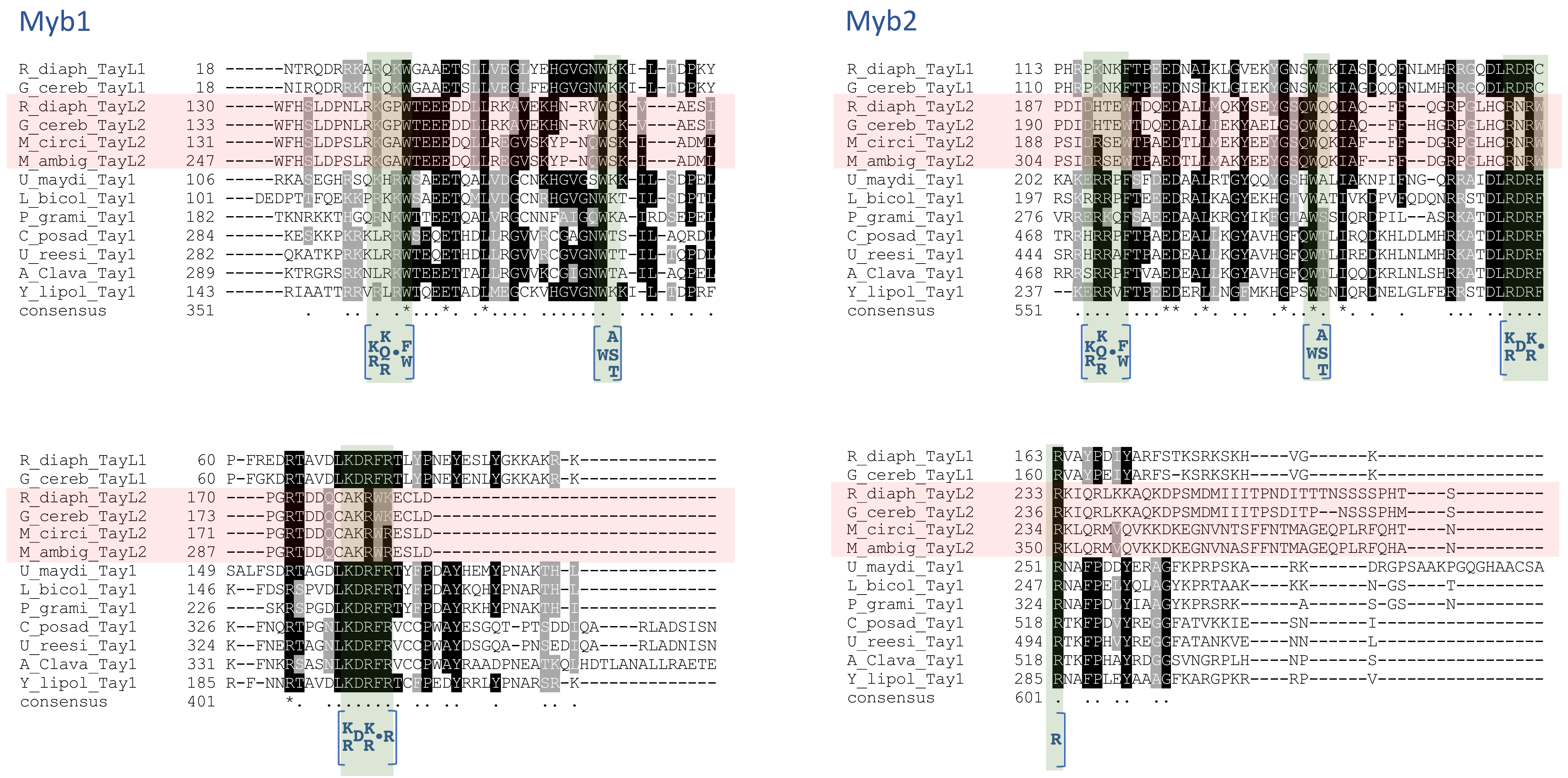

Supp. Fig. 1 Sequence alignments of Tay1, TayL1, and TayL2 family members

The Myb1 and Myb2 motifs for Tay1, TayL2 and TayL2 family members are aligned using T-coffee. The conserved motifs in TRF1 family members involved in binding the TTAGGG repeats (based on crystal structures in Court et al., 2005) are displayed below the alignments. The proteins included in the alignments are as follows: R_diaph_TayL1, from Rhizophagus diaphanous, RGB43698.1; G_cereb_TayL1, from *Glomus cerebriforme*, RIA97975.1; R_diaph_TayL2, from *Rhizophagus diaphanous*, RGB438099.1; G_cereb_TayL2, from *Glomus cerebriforme*, RIA96913.1; M_circi_TayL2, from *Mucor circinelloides*, EPB89450.1; M_ambig_TayL2, from *Mucor ambiguus*, GAN08276.1; U_maydi_Tay1; from *Ustilago maydis*, Um02326.1; L_bicol_Tay1, from *Laccaria bicolor*; XP_001886326; P_grami_Tay1, from *Puccinia graminis*, XP_003325585; C_posad_Tay1, from *Coccidioides posadasii*, EFW18452; U_reesi_Tay1, from *Uncinocarpus reesei*, XP_002540930; A_clava_Tay1, from *Aspergillus Clavatus*, XP_001270706; Y_lipol_Tay1, from *Yarrowia lipolytica*, XP_502676.
